# Exploring provider and community responses to the new malaria diagnostic and treatment regime in Solomon Islands

**DOI:** 10.1186/1475-2875-10-3

**Published:** 2011-01-10

**Authors:** Rushika S Wijesinghe, Jo-An M Atkinson, Albino Bobogare, Lyndes Wini, Maxine Whittaker

**Affiliations:** 1 The University of Queensland, School of Population Health, Pacific Malaria Initiative Support Centre, Australian Centre for International and Tropical Health, Brisbane, Australia; 2 National Vector Borne Disease Control Programme, Ministry of Health, Honiara, Solomon Islands

## Abstract

**Background:**

Improvements in availability and accessibility of artemisinin-based combination therapy (ACT) for malaria treatment and the emergence of multi-drug-resistant parasites have prompted many countries to adopt ACT as the first-line drug. In 2009, Solomon Islands (SI) likewise implemented new national treatment guidelines for malaria. The ACT, Coartem^® ^(artemether-lumefantrine) is now the primary pharmacotherapy in SI for *Plasmodium falciparum *malaria, *Plasmodium vivax *malaria or mixed infections. Targeted treatment is also recommended in the new treatment regime through maintenance of quality microscopy services and the introduction of Rapid Diagnostic Tests (RDTs). Ascertaining the factors that influence community and provider acceptance of and adherence to the new treatment regime will be vital to improving the effectiveness of this intervention and reducing the risk of development of drug resistance.

**Methods:**

In order to understand community and prescriber perceptions and acceptability of the new diagnostic and treatment interventions, 12 focus group discussions (FGDs) and 12 key informant interviews (KII) were carried out in rural and urban villages of Malaita Province, Solomon Islands four months subsequent to roll out of these interventions.

**Results:**

Lack of access to microscopy or distrust in the accuracy of diagnostic tools were reported by some participants as reasons for the ongoing practice of presumptive treatment of malaria. Lack of confidence in RDT accuracy has negatively impacted its acceptability. Coartem^® ^had good acceptability among most participants, however, some rural participants questioned its effectiveness due to lack of side effects and the larger quantity of tablets required to be taken. Storing of left over medication for subsequent fever episodes was reported as common.

**Conclusion:**

To address these issues, further training and supportive supervision of healthcare workers will be essential, as will the engagement of influential community members in health promotion activities to improve acceptability of RDTs and adherence to the new treatment regime. Exploring the extent of these issues beyond the study population must be a priority for malaria programme managers. Practices such as presumptive treatment and the taking of sub-curative doses are of considerable concern for both the health of individuals and the increased risk it poses to the development of parasite resistance to this important first-line treatment against malaria.

## Background

Since first proposed by the World Health Organization in the early 1990s, the strategy of early diagnosis and prompt effective treatment of malaria illness has been a cornerstone of malaria control [1,2]. In recent times, the Roll Back Malaria Partnership has set the target for 2010 of "80% of malaria patients to be diagnosed and treated with effective anti-malarial medicines within one day of the onset of illness" [3]. To achieve this, many interventions have been developed and strategies employed to improve accuracy of diagnostic testing and reporting of results. This has the additional benefit of reducing the waste and cost of anti-malarial medication and improving treatment of alternative causes of fever [3,4]. It has also been recommended that programmes aim to build health system capacity to increase coverage of services and improve the prescription and dispensing practices of providers [5].

Malaria is the predominant cause of febrile illness and a major public health problem in Solomon Islands (SI) [6]. In 2009, there were 40,136 reported malaria cases in the country, with an annual incidence rate of 77/1,000 population, of which *Plasmodium falciparum *accounted for 72% and *Plasmodium vivax *28% [6]. There were 13 deaths due to malaria in 2009. However, malaria hospital admissions showed a significant decline in 2009 compared to the two previous years and there is evidence of a gradual decline in malaria cases over the period 2001 to 2009. There has also been a general decline in malaria annual incidence rates over the period of 2003 - 2009 [6]. Malaita Province, where the current study was carried out, had a malaria incidence rate of 82.9/1,000 population in 2009, which was amongst the top three provinces for malaria incidence reported in that year [6]. In 2008, SI raised the goal of their National Malaria Programme (NMP) to intensified control and progressive elimination. In line with the current global strategy, a key component of the programme is capacity building for early diagnosis and prompt and effective treatment [7].

Concerns about emerging resistance to the previously existing regime of chloroquine and sulphadoxine-pyrimethamine (Fansidar^®^), prompted a review of the management of malaria in SI and subsequently, the new national treatment guidelines for malaria were implemented [8]. The new drug regime recommends artemisinin-based combination therapy (ACT) with artemether-lumefantrine (Coartem^®^) as first-line pharmacotherapy for *P. falciparum *malaria, *P. vivax *malaria and mixed infections. The new regime also recommends treatment after definitive diagnosis and aims to reduce presumptive treatment. This can be achieved by increasing diagnostic capability of peripheral-level health services either through use of good quality microscopy or through the introduction of Rapid Diagnostic Test (RDT) kits [8].

RDTs can be used as a stop-gap when microscopy services are not operating (e.g. evenings/weekends/public holidays) or as a primary diagnostic tool for rural/remote areas without microscopy services [8]. Malaria RDTs have being suggested to improve diagnostic efficiency, which is important for preventing indiscriminate use of ACT, thereby preventing or delaying the development of parasite resistance to this new first-line drug [9]. Several studies have shown that using RDTs to direct the use of ACT has the added advantage of allowing more accurate treatment of fever, which is important for improving patient care and minimizing costs for malaria control programmes [9-13]. A study in Zanzibar in 2009 found the introduction of RDTs resulted in improved management of patients presenting with fever without increasing cost per patient, leading authors to suggest RDTs as an important tool for improving fever management in peripheral health care settings [9].

Furthermore, Lubell *et al *[11] found that at moderate and low levels of malaria transmission in Tanzania, RDTs were more cost beneficial than microscopy, and both more so than presumptive treatment, but only where treatment response was consistent with test results. In cases where prescriptions of anti-malarial drugs were given to patients with negative tests results, neither microscopy nor RDTs were found to be cost beneficial. Depending on transmission rate, these authors reported that the cost of treatment inconsistent with diagnostic outcome was 10 - 250% higher than treatment that was consistent with test results [11]. Investment in building diagnostic capacity to minimise indiscriminate presumptive treatment with ACT will be considerably less costly than dealing with the consequences of the development of parasite resistance to this important first-line treatment against malaria [12].

Realizing the potential of RDTs and ACT for intensified malaria control and elimination depends upon provider and community response to and utilisation of these new tools for diagnosis and treatment. Acceptability of RDTs and ACT can be affected by perceptions and misconceptions of their accuracy, safety and effectiveness. Studies carried out in Tanzania and Uganda found that despite recognising their potential to improve clinical diagnosis, RDTs were suspected by the community of being used to test for HIV (Human Immunodeficiency Virus) status, that they could infect children with HIV and that the blood samples could be used for witchcraft [14,15]. Community acceptability of RDTs was also found to be influenced by the education level of healthcare workers which affected community confidence in their expertise [15]. Furthermore, healthcare worker non-adherence to RDT use has been found to be a consequence of inconsistencies in training messages, insufficient technical supervision and inadequacies in logistical support for their consistent supply [15].

Factors affecting healthcare worker prescription of ACT have been reported to include patient tolerance of the drug, its effectiveness, adequate drug supply and staffing, training messages that are consistent with recommended guidelines and follow-up supervision [16]. Factors influencing community acceptability of ACT are suggested to be inter-related and include provider-patient relationship and trust in the quality of care, patient expectations, beliefs regarding illness aetiology and perceived effectiveness of treatment [17]. These issues can have a significant impact on patient adherence to the ACT treatment regime. A study carried out in Sierra Leone found as little as 48.3% of patients had probable adherence to artesunate and amodiaquine therapy provided through community health centres [18]. This was attributed to side effects after the first dose, food not being available to accompany drug intake, forgetting to take doses and incorrect instructions given by the health centre [18].

It is vital to evaluate local factors that influence RDT and ACT acceptability and use early in the post-implementation period in order to rapidly address community and provider concerns and misconceptions. This would prevent issues of mistrust of interventions and of healthcare workers which can otherwise result in a costly waste of valuable resources and more critically, can undermine malaria control and elimination programmes. This qualitative study was, therefore, carried out to investigate community and prescriber perceptions and acceptability of the new diagnostic and treatment interventions in SI. The results will be used to inform quality improvement of the newly implemented treatment regime and to assist with planning for national scale-up of RDTs and ACT in SI.

## Methods

### Study area and target population

**Figure 1:**
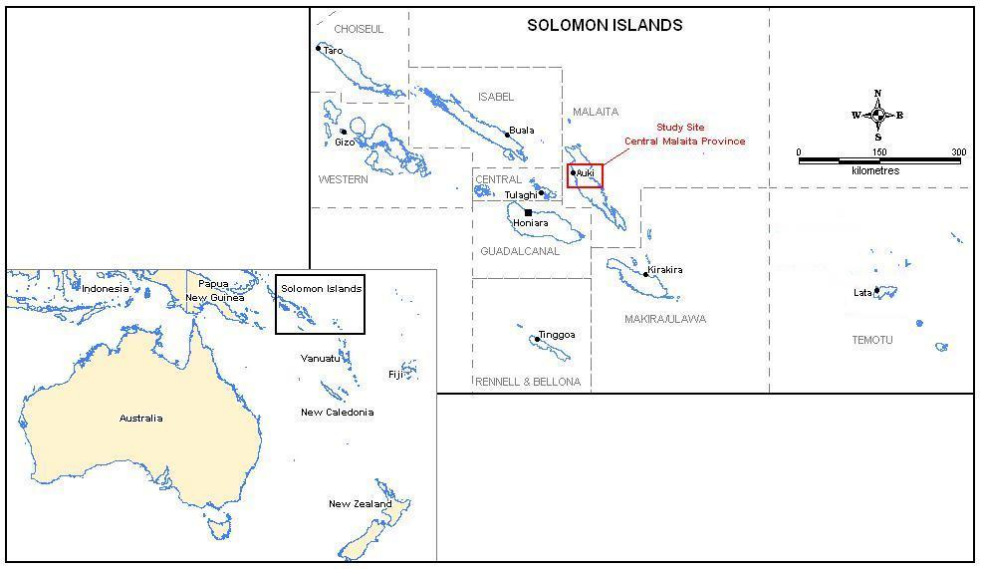
**Map of location of study site**.

This study was conducted in March 2010 in Malaita Province, SI (Figure 1). This Province was purposefully selected as it has one of the highest incidences of malaria in SI [6], and hence intensified control activities there had included training of healthcare workers and roll-out of RDTs and ACT four months prior to commencement of the study. Designed as an exploratory study, in-country public health and research officers guided the selection of one rural and one urban village within one hour of the Provincial Capital, Auki, in order to capture potential differences in provider and community attitudes and perceptions. A convenience method was used to recruit study participants from village church groups, womens' clubs and youth groups in consultation with local village leaders.

### Procedure

The field work was carried out by male and female contracted local research officers of the Solomon Islands Development Trust (SIDT), supported and supervised by staff of the National Vector Borne Disease Control Programme (NVBDCP), Ministry of Health, SI and the Pacific Malaria Initiative Support Centre (PacMISC) at the School of Population Health (SPH), University of Queensland (UQ), Australia. The SPH team have several years of experience in Melanesia including SI and have been working very closely with the local co-investigators of the NVBDCP who are intensively involved in the malaria programme in the country. A four-day workshop was conducted by SPH staff to train the field research team in qualitative research methods, research logistics, ethical considerations, equipment use and data processing.

Focus Group Discussions (FGDs) were carried out separately with men, women and youth (mixed gender) groups in order to facilitate open, unrestricted dialogue. Key Informant Interviews (KIIs) were conducted with healthcare workers and other community leaders in each village such as chiefs, teachers, womens' group activists and religious leaders. Interviews were carried out in Solomon Islands Pijin, were facilitated by a field research officer and supported by a local language interpreter as required. Semi-structured interview guides were used to direct discussions. Interviews were recorded with consent using a digital tape recorder, backed up with manual note taking. Demographic data of participants was also obtained including age, education, occupation and religious affiliation.

Throughout the study, there were regular consultations between the field research staff and the staff of SPH and NVBDCP. This included the SPH team reviewing a sample of early transcripts and their translations when the research was underway to identify any issues in quality, method, translation and misinterpretation of interviews guides. Advice and feedback was given and changes made accordingly. An additional aim of the study was to continue capacity building in SI by training local staff in qualitative research methodologies; therefore comprehensive feedback was provided to them during and after the work was completed.

### Data analysis

In-depth analysis was carried out by the SPH research team at the University of Queensland. Digital recordings of the FGDs and KIIs were directly transcribed and translated from Solomon Islands Pijin (or local dialect) to English by the field research team. Data was coded around the main topics of the interview guides and entered into NVivo 8 software (QSR International Pty Ltd, Australia). The SPH team developed an agreed coding key through sample transcript reviews and discussions of coding issues, which was then utilised by the primary coder. The data was organized into identifiable themes and patterns of behaviour and subjected to thematic analysis. Areas of consensus and divergence were identified and a 'realist method' was used to understand participants' realities, experiences and meanings. This approach has previously been reported to be appropriate for working within a participatory paradigm particularly where research findings are informing policy development [19]. Discussions were held throughout the coding and analysis process with the SI co-investigators for clarification, back translation if required and confirmation.

### Ethical aspects

Ethical approval for this study was obtained from the Solomon Islands National Health Research Ethics Committee and the University of Queensland Behavioural and Social Sciences Ethical Review Committee. Individual informed consent (written or witnessed thumb print) was obtained from all participants prior to the FGDs and KIIs following a verbal and written explanation of study aims and procedures. The privacy of participants was preserved as the transcripts did not identify individuals by their full names. Participant information (digital recordings from FGDs, KIIs and records of participant demographic details) was securely stored in the field and subsequently in Honiara and Brisbane.

## Results

In total, 12 FGDs and 12 KIIs were carried out with 135 participants from urban and rural villages in Malaita Province over four weeks. The number of participants in each FGD ranged from 7 to 11. The age range for male participants was 22 - 80 years, for females 23 - 56 years and for youth, 16 - 18 years. Participants were primarily members of the South Seas Evangelical Church.

### Treatment-seeking

Treatment-seeking practices reported by men, women and youth were similar; however, differences emerged when comparing responses in urban and rural villages. Participants in the urban village were more likely to identify the importance of early treatment-seeking for adults with fever and reported accessing either the local nurse aid post or Provincial Hospital within 12 to 24 hours of onset of symptoms. Participants in the rural village reported waiting 2-4 days post onset of fever before accessing the aid post. The reasons for this difference are unclear; however, a few participants reported that the distance to the aid post was sometimes a prohibiting factor and one participant perceived that malaria may not be correctly diagnosed if he attended the clinic soon after onset of fever.

*'I first observe the symptoms of malaria until it becomes more obvious so that when I go for a test, they straight away find malaria, because, in some cases when I went early to get a test (for malaria), my result is often negative. So I have to wait until I notice the symptoms becoming more obvious before I go for a test and after the test my result confirms malaria.' *(male participant, FGD urban village)

Some participants in both rural and urban villages reported that due to their vulnerability, children and pregnant women with fever should go to the clinic or hospital without delay to be tested and treated. Many in the rural village, however, stated that the usual practice even for these vulnerable groups was to wait until the fever becomes more serious (waiting up to four days) prior to attending the aid post or hospital. Until that decision point, a carer/family member of the patient themselves would manage the fever at home, typically voiced by this male participant;

*'...(people) will commonly wait until (a mother or child) gets seriously sick before they will take them to the clinic. This is what usually happens in rural areas....when a child has a fever, I will treat them with the leftover medicine at home for three days and then if they don't recover I will take them to the clinic.' *(male participant, FGD rural village)

Home treatment (reported by most participants in both urban and rural villages) consists primarily of giving a person with fever paracetamol or aspirin and sponging them with cool water. Some participants in both villages reported using left over malaria medication if they had it at home. This was used if they suspected malaria before they attended a clinic or if the clinic did not diagnose malaria but the participants believed the diagnosis to be in error. Some less commonly reported home remedies for fever included steaming, drinking lemon juice, eating lots of fruit, taking custom medicine and/or praying for the sick person.

### Experiences of presenting with fever to an aid post

Most participants in the urban and rural communities reported that when they present to an aid post with fever, a blood slide is usually taken to test for malaria. Treatment was commonly reported as directed by microscopy results. If the test is positive they are given malaria treatment, if the test is negative they are given paracetamol, aspirin or 'Septrin^®^' (cotrimoxazole, an antibiotic). Some participants in both villages also reported that if a microscopist is not available or if a significant delay in obtaining results is expected, then presumptive treatment for malaria is given by the nurse on the basis of presenting symptoms. A few participants and a key informant in the urban village also reported that on occasions the nurse at the aid post provides presumptive treatment if they suspect malaria even when the microscopy results are negative, as expressed by a male participant;

*'...but now if you are positive or negative (for malaria) they will give you Coartem^® ^tablets.' *(male participant, FGD urban village)

The issue of lack of patient confidence in accuracy of microscopy and nursing staff was raised by a few participants in both urban and rural villages. These participants highlighted that a lack of trust in diagnostic accuracy usually results in demands for presumptive malaria treatment by the patient, cross checking of results at another clinic (or with a doctor at the Provincial Hospital) or taking of left over malaria medication they are able to obtain back at their village.

*'...some people usually force the nurse to give malaria treatment even if test result (microscopy) is negative, but if a doctor tells them (that they don't have malaria) they will accept treatment with Panadol^® ^or Septrin^®^.' *(male participant, FGD urban village)

*'Sometimes a parent will bring their child (to the clinic) and when the test result (microscopy) is negative they come back to the village and give leftover (malaria) medication to the child to drink...' *(female participant, FGD urban village)

'*If my child was sick with a fever....and the woman does not have any malaria treatment at home, they go around the village and ask people if they have any malaria treatment.' *(male youth participant, FGD rural village)

One male participant from the rural village suggested the potential negative impact of presumptive treatment for malaria by highlighting that if the fever was caused by another illness, the malaria medication will not work. Dissatisfaction with the nurses' diagnosis of the cause of fever was highlighted by a few participants in each of the FGDs carried out with men and youth of the rural village.

*'...a few weeks later I felt sick again so I went to the clinic and they told me my test was negative, but I know I had malaria...so I forced the nurse to give me malaria treatment. But some nurses, if they see the result is negative they will not give me malaria treatment, they would just give me Panadol^® ^and aspirin and then they justify it by saying that the cold or fever is hidden in your lung. I am not sure whether they are telling the truth or not....this happened to me and I am not happy.' *(male participant, FGD rural village)

### Healthcare worker perceptions of RDTs

None of the healthcare workers interviewed claimed they had received any recent formal training in the use of RDTs. All healthcare workers interviewed reported their lack of confidence in the accuracy of RDTs. Specifically, they reported a concern that RDTs were not sensitive enough to detect malaria when there is low parasite density in the blood. Negative RDTs are usually cross-checked with microscopy particularly for pregnant women or children. When microscopy is not available to confirm the negative RDT results, many healthcare workers reported giving presumptive treatment if they suspected malaria. Lack of training in the proper use of RDTs did not appear to be the only reason for a lack of confidence in the test results as reflected in these typical comments by many of the healthcare workers.

'*RDT can detect the high fever but the lower fever it doesn't detect.'*

(healthcare worker 1, KII, urban village)

*'...for hard cases to do with Pv, given that the protozoa are so tiny, they need cross checking (with microscopy) especially for pregnant mothers and children or middle age group.' *(healthcare worker 1, KII, rural village)

*'During the first introduction of RDTs, I was involved in using them for some time but one thing I found out is that (the RDT) is not as accurate as we would expect. We found that the same (blood sample) that was negative in RDT, is positive in microscopy. So now we don't use RDT.' *(healthcare worker 3, KII, urban village)

Other commonly reported disadvantages of RDTs were their variable and often insufficient supply as well as their inability to visually detect the parasite and therefore support prescribing of treatment.

*'(Microscopy) is always available here because we have power, but for RDTs, when it runs out of stock we have to wait for a long period of time before we have it in our stock, whereas microscopy is always available.' *(healthcare worker 1, KII, urban village)

*'I prefer microscopy because it is visual where I can see the parasite and identify the real parasite, not like RDTs where there is only chemical detection of the parasite. I don't really trust the RDT.' *(Healthcare worker 2, KII, urban village)

Despite these perceived disadvantages, RDTs were commonly recognized as being potentially useful in rural and remote areas where there is no microscopy service as well as in urban and less remote rural clinics for use on weekends and evenings when the microscopist is not available. RDTs were also reported as being provided free of charge to patients (compared to microscopy which reportedly incurs a charge to patients), provides faster results and were identified as useful when there is no power to use electric microscopes.

### Community perceptions of RDTs

None of the participants in the urban village and only a few participants in the rural village (primarily the women) reported knowing what RDTs are or had experienced this test. Once this test was briefly explained to participants they reported their perceptions of the potential advantages and disadvantages. Most participants in both villages reported the advantage of the RDT as its ability to provide prompt test results. A few participants also identified that they were useful in poor weather when community microscopes are not operational and that they are a way of avoiding human error. For these reasons, some participants in both villages indicated perceived acceptability of RDTs.

Despite these advantages and nominal acceptability expressed by some participants, in most interviews participants, particularly those that had experienced their use, expressed their doubts regarding the accuracy of a 'rapid' test. These doubts would reportedly prompt demands for presumptive malaria treatment, crosschecking with microscopy (through attendance at multiple clinics) or the taking of left over malaria medication that may be available at their home or village.

*'I think I would doubt this new test (RDT) because it is very fast and might not properly diagnose the blood, whereas the microscope takes time for diagnosing and might be more accurate than the new test.' *(male participant, FGD urban village)

*'I was uncertain whether it gave a true result because at least with the microscope they actually look at the blood. I think this is the true one, because it takes a long time and they look at the blood.' *(female participant, FGD rural village)

The lack of trust expressed in RDT and in negative results of microscopy appears to arise from the incongruence between the diagnosis provided by the aid post nurse and the patient's self diagnosis of malaria and expectations for malaria treatment. The majority of the community members strongly voiced their expertise in clinical diagnosis of malaria given their history of experience with this disease.

*'...my expectation is that my child will be positive but instead she was negative so I suggest that this RDT gave a false result so I don't trust this sort of test.' *(male participant, FGD rural village)

*'Almost everybody in every house knows how to diagnose themselves of which sickness they have, whether it is malaria or other diseases.' *(male participant, FGD urban village)

### Healthcare worker perceptions of ACT

Healthcare worker attitudes to Coartem^® ^(artemether-lumefantrine, AL) differed between the urban and rural village. Healthcare workers in the urban village reported that AL has better acceptability than chloroquine amongst their patients, there are fewer complaints and there has been better compliance with completion of the full dose as it has no side effects. Although some criticisms included its occasional lack of supply and that it is not available as an injection for the treatment of more severe malaria cases, overall AL has good acceptability among healthcare workers in this village and they perceived a reduction in the number of malaria cases since its introduction.

*'Yes...we have seen change take place, that is, the number of cases of malaria has decreased due to the introduction of Coartem*^®^. *Not like before during the time of chloroquine where we had a lot of positive cases. In one day we now only see 60 slides whereas before in one day we usually read 200 slides.' *(healthcare worker 2, KII urban village)

In contrast, healthcare workers in the rural village reported problems with patients complying with the timing of doses (i.e. some forgetting to take evening tablets) of AL and one aid post worker reported that it is less effective than chloroquine (with anecdotal reports of AL resistance based on poor response of presenting symptoms to the AL treatment). Due to these reasons, one healthcare worker reported that some of his patients will no longer accept AL for malaria treatment.

*'...the problem patients have is to drink the medicines twice a day. If they don't have a watch they just take the medicines anytime they feel they want to take it*.....*at least 10% of the population of the catchment area I look after don't want Coartem^®^. After taking the medicine they would come back and check and they still have malaria. So for this reason they don't want Coartem^®^.' *(healthcare worker 2, KII rural village)

### Community perceptions of chloroquine

Use of chloroquine was frequently mentioned by patients, and was often compared with the new drug *Coartem*^®^; therefore these perceptions were recorded and analysed, even though chloroquine is not part of the new treatment regime. Perceptions and experiences with malaria medication did not appear to differ between the rural and urban village nor by gender or age. Chloroquine was the most well-known and commonly used malaria treatment reported among participants. Although some participants in both rural and urban villages stated the importance of completing an entire dose of chloroquine provided by the nurse or doctor in order to cure malaria, these participants were mostly those that held positions of leadership in their communities.

*'It is tambu (forbidden) to give the medicine to anyone. If I don't complete my dose the sick will continue.' *(village elder, KII rural village)

Reasons provided by the majority of the participants for poor adherence to the chloroquine dosage regime were the bitter taste, experience of other side effects (commonly reported were body itchiness and dizziness) or the habit of ceasing its use once malaria symptoms are relieved.

*'...I usually experience body scratch so I just stop taking (chloroquine) and put the rest aside. If I notice this feeling following the first dose, I will straight away put aside the rest of the tablets.' *(male youth participant, FGD urban village)

Less commonly reported side effects were abdominal discomfort, weakness and tiredness.

*'Another thing about that medicine (chloroquine) is it affects your body and makes it weak, by the time it comes to the second day you feel like you are drunk so I don't finish the medicine.' *(male youth participant, FGD rural village)

Despite these side effects, chloroquine was perceived to be effective by most participants. Chloroquine tablets not used were commonly reported to be kept by households for subsequent episodes of fever for themselves, other family members (including children) or occasionally for others in their community.

*'I stop taking the tablets (chloroquine) when I start to get better...The left over medication I keep in the house for next time if I get sick...or for some time when family come and ask for medicine I will give it to them.' *(female participant, KII urban village)

*'If I had left over medicine I would keep it, because sometimes it is hard to get to the clinic, so you need to keep some medicine.' *(male youth participant, FGD rural village)

### Community perceptions of ACT

Some participants in both rural and urban villages reported having taken the recently introduced ACT, *Coartem*^® ^(artemether - lumefantrine, AL). Good acceptability was reported by most of those who had used AL due to its effectiveness, the lack of side effects and its pleasant taste. It was also perceived to be safe to use for pregnant women and children if prescribed and provided by a healthcare worker.

*'This one (Coartem^®^) is nice. It wins over the old one, it tastes sweet and it is easy to take.' *(female youth, FGD rural village)

*'...so after I completed 2 treatments (Coartem^®^), I felt better and I left out the last treatment...I found its effect to be faster than chloroquine.' *(male participant, FGD urban village)

A few participants of the male and youth FGDs in the rural village however, perceived the lack of side effects of AL and the larger quantity of tablets needing to be taken as evidence of its potential ineffectiveness or lack of strength.

*'The old one was 12 tablets and the new one is 24 tablets. You have to take four in the morning and four in the evening. This is three times more than the old type of pill. I am worried; do the new ones have similar strength for curing malaria as the old ones?' *(male participant, FGD rural village)

*'I like the old treatment because I know when it makes me feel drunk that the treatment is so strong so it must be working. According to the new one, you have to take 8 tablets for three days but you still never feel drunk, so the medicine must be weak/ineffective.' *(male participant, FGD rural village)

Reports of storing of left over AL in households for subsequent fever episodes emerged during the discussions with a few participants admitting to have done this themselves. However, due to differences between packaging (loose tablets are provided for chloroquine and pre-packaged blister strips for AL) one participant reported the storing of AL to be more difficult than chloroquine.

*'It is important that when you open the packet of the new tablets (Coartem^®^) that you take them straight away so they are not exposed to the air, otherwise the strength is reduced. But the old medicine is different; you can just keep the left over tablets in the bottle.' *(male participant, FGD rural village)

### Participant recommendations

Most participants (both healthcare workers and villagers) recommended that further awareness be carried out to improve community knowledge of the new diagnostic and treatment interventions. Many of the village participants suggested that this awareness will most effectively be carried out by healthcare workers or trained volunteers through regular visits to communities as this will allow villagers to receive education, ask questions and have their concerns addressed.

*'They should set up programs to come down to the community to provide awareness and health service to the village people so that they will not find it difficult to go to the clinics.' *(youth participant, FGD urban village)

*'...They must show the community how to use the treatment. People have a lot of questions. They must explain the medicines well...In order to clear peoples' minds there is a need for nurses to go around and talk to people rather than people only being able to ask questions when they come to the clinic only.' *(elder, KII rural village)

Particularly in the rural village, some participants requested education regarding the different types of malaria, why the new drug AL is being introduced and when and how it should be used.

*'The nurse did not come to the village to explain. We don't know why we need to use the new treatment not the old treatment. How come? Is it because it is bad? They need to come to the village and explain why we need to take the new one.' *(male participant, FGD rural village)

## Discussion

This study was carried out to investigate community and prescriber perceptions and acceptability of the new diagnostic and treatment interventions in SI. Determining malaria status has been advocated as an important tool for reducing overuse of the more expensive ACT, to improve the management of non-malarial febrile illness and to prevent emergence of parasite resistance to this new first line drug [9]. However, the use of RDTs has elucidated a number of human behavioural challenges which can negatively impact the acceptability and use of RDTs and jeopardize the cost-effectiveness of their implementation.

### Lack of healthcare worker confidence in RDT use

Healthcare workers in the current study reported inadequate training in the use of RDTs. Although a pre-rollout training program for Provincial healthcare worker representatives did occur in the SI capital (Honiara), the 'cascade training' system implemented by the malaria programme had not reached many of the healthcare workers of the villages that participated in this study. This approach to training refers to the dissemination of knowledge through multiple levels until it reaches peripheral healthcare workers, but has been criticized as being ineffective due to the dilution of key messages and its lack of continuation [20]. A previous study on the effectiveness of 'cascade training' has shown that although initially it is more cost-effective and can provide some improvements in quality of care and access, it had less impact than supportive supervision to reinforce guideline dissemination over time [21]. The extent of Provincial-level training of healthcare workers in RDT use is not known and hence it is recommended that this be reviewed prior to national scale-up of this intervention.

Manufacturer's instructions for RDT use have been found to be insufficient to ensure their safe and accurate use [22]. Given that the accuracy of RDT results can depend on factors such as storage and end-user performance, it is vital to implement an adequate training system and provision of ongoing supervision, technical/logistic support and feedback of quality control results for RDTs [22-24]. Significantly higher accuracy in using RDTs have been demonstrated in a Zambian study through the use of pictorial instructions (kept for reference as a 'job aid') and a brief training session (3 hours) when compared to the use of manufacturer's instructions alone [22]. Other suggested content for a training curriculum for RDTs are unambiguous guidelines, training in alternative causes of disease and strategies to improve healthcare worker - patient relationships [24]. As well as ensuring accurate results, adequate training can help foster healthcare worker confidence in the use and results of RDTs and improve their acceptability by the community, which may reduce the practice of presumptive treatment for malaria using ACT [15,22].

### Lack of trust in negative diagnostic results

In this study, suspicions of inaccuracy of negative results of RDTs by both healthcare workers and community members were found to adversely affect their acceptability and result in practices of confirming results with microscopy, provision of presumptive treatment or the taking of un-prescribed left-over medication available in the community. These practices, if widespread, have the potential to significantly undermine the cost-benefit of RDTs as an adjunct or alternative to microscopy in SI [11].

Although double checking of results was reported more often with the use of RDTs in this study, microscopy results were also questioned by some community members and would result in similar practices of double checking of results, self-medication or demands for presumptive treatment. This seemed to occur when their self-diagnosis was inconsistent with the diagnostic conclusions made by the healthcare worker and hence their expectations of service were not met. Additionally in SI, community members are familiar with microscopy examination of blood smears taking some time to "heat the blood and look at it" when diagnosing malaria. The lesser duration taken for diagnosis by RDT needs more careful explanation to the community both at the time of consultation, as well as at community level health promotion to facilitate its acceptability. The sustainability of ACT use as a first-line drug will be threatened if these practices cannot be sufficiently addressed [25].

Previous studies carried out in Tanzania have indicated that approximately 50% of patients who were negative by microscopy or RDTs were prescribed antimalarial drugs [25,26]. It is uncertain from the current study the extent of over-prescription of anti-malaria medication in SI. In addition, it is unclear as to whether presumptive treatment is occurring as a result of community expectations and hence pressure to prescribe or due to a lack of healthcare worker trust of negative diagnostic results or both. Therefore, the SI National Malaria Program will benefit from addressing the issue from both perspectives.

Patients living in malaria endemic areas that have experienced multiple episodes and recognize their key physical symptoms as malaria are less likely to accept a negative RDT result. Travelling further to seek additional testing using a more sensitive diagnostic tool or retesting using an RDT by another healthcare worker some time after the initial test could potentially result in a positive diagnosis for malaria. This process, which was described by participants of the current study, could significantly undermine the confidence community members have in the expertise of their local healthcare workers, due to their lack of understanding how RDTs work and how changing levels of parasitaemia over time can affect the results. Whilst health promotion activities should focus on encouraging early diagnosis and treatment, it should also reiterate that a negative RDT result could potentially occur due to low parasite densities at the early stage of the disease. Therefore, patients should also be advised to return to the health facility for a repeat RDT/microscopy if symptoms do not resolve, or worsen. In designing messages to address this in SI, care must be given not to contradict messages encouraging early treatment-seeking for fever management.

Studies carried out in Africa have shown that patient demands for presumptive malaria treatment were less likely to occur where the community had confidence in the education level of community healthcare workers using RDTs [15,27]. In SI, particularly at the peripheral level, there are very few physicians, with the majority of health service providers being nurses and nurse aids. Community acceptability and trust in diagnostic results as well as confidence in their healthcare workers' clinical diagnostic capabilities will need to be enhanced in SI. This can be done through a broader health system strengthening approach that includes adequate training in diagnosis and management of a range of febrile illnesses. This will need to be followed up with regular supportive supervision.

### Challenges relating to the use of ACT

Most malaria endemic countries in Africa and Asia have now adopted ACT into their anti-malarial drug policy [28]. However, in order to maximize their effectiveness, behavioural factors affecting early and complete treatment need to be addressed. In the current study ACTs were well accepted and reported as favourable by most community and healthcare workers interviewed. Their acceptability was primarily a result of their lack of side effects when compared to chloroquine. Similar findings were noted in a study investigating community acceptability and perceptions of ACT in Tanzania [29]. Despite their high acceptability, delays in treatment-seeking and non-completion of doses, both reported in our study as well, can limit the impact of the drug and increase the risk of resistance emerging [29]. However, there were also concerns expressed in our study population that ACT is not strong enough. Ironically, the side effects complained regarding chloroquine are also perceived by Solomon Islanders as evidence of them being strong enough to rid the body of malaria and that they are working effectively. The bitterness of chloroquine, a characteristic of traditional medicines used to treat malaria in SI, is a sign of efficacy for malaria treatment, transmitted verbally through generations.

Delays in treatment-seeking (of 2-4 days) were primarily reported by participants in the rural village. The reasons for this remain unclear; however as noted, one participant discussed differences in the accessibility of services as a cause. Alba *et al *(2010) reported in their study in the rural areas of Ifakara, Tanzania that the availability of outlets (health facilities or drug shops) was the most important determinant of whether patients receive prompt and effective treatment, whereas affordability and accessibility contributed to a lesser extent [30]. Similarly, in the poorest areas of four malaria endemic districts in Kenya, facility opening hours, organisation of health care services and staff shortages were identified as barriers for availability to prompt and effective treatment [17]. Importantly, in the rural community of the current study, an increase in the severity of fever seemed to signify malaria and motivate treatment-seeking. This misconception, combined with reports of less convenient access to a healthcare worker, lack of confidence in healthcare workers' diagnosis of fever and the availability of left-over medication in the village appeared to be the barriers to early treatment-seeking in this rural village.

A few participants including a healthcare worker reported poor acceptability of ACT resulting in some refusals, as they were perceived to lack strength and effectiveness compared to chloroquine. This may be a result of reported problems with adherence to the dosage regime or incorrect presumptive malaria treatment for a non-malarial fever. Although reported by only a few participants, these issues highlight the negative impact human behavioural factors can have on the success of malaria control interventions, particularly in neglected rural communities.

A concerning practice described in the study populations was the sharing of malaria medicines within the family or from other community members. This was likely more easily done when medicines were provided in loose form compared to needing to physically "pop" the ACT from the blister packs in which they are provided. Although this practice is concerning regardless of the type of medication that is shared, Coartem^® ^is specifically weight/age prescribed and packaged and also sensitive to deterioration if removed from the packaging. The need to focus on informing the community of these concerns and of the rationale for correct use of prescribed medicines is highlighted by these findings.

Another issue raised in the study are the reported difficulties by community members in taking Coartem^® ^according to the recommended dosing regimen (i.e. one dose immediately, second dose after 8 hours, then every 12 hours for the rest of the three-day course). Conveniently, the dosing regimen of Coartem^® ^is similar to Septrin^® ^which is a widely used and familiar antibiotic in SI. Healthcare workers could therefore harness this existing knowledge to guide patients in completing the new ACT dosing regime.

The National Malaria Programme in SI has recognized the need to ensure that the central level health staff who are influential both as educators for nurses and nurse aides as well as community role models must be convinced of and support the new treatment regime. In the early stages of the introduction of the new treatment regime there were anecdotal reports that senior nurses at the National Referral Hospital were continuing to use chloroquine as they had more confidence in this drug. Special efforts have since been made to convince influential health staff to become advocates for the change. It is hoped that this will improve confidence in the use of the new treatment regime by both peripheral health staff and their communities.

Ensuring that all individuals suffering from malaria have prompt access to effective treatment remains a challenge for resource constrained health systems [17]. Nevertheless, the potential public health benefits of the introduction of free, efficacious ACT in SI will not be realized if provider prescription practices do not conform with the recommended guidelines or if patients do not adhere to treatment regimes or self-medicate with sub-curative doses. Through a number of qualitative and quantitative evaluations, the SI National Malaria Program has demonstrated their commitment to regular monitoring of human behavioural factors that impact intervention acceptability and use. Community feedback provided through such investigations contributes to informed policy decision-making and are integral to the success of the program. Based on the results of this study, the SI National Malaria Programme is planning similar investigations into community and healthcare worker perceptions and attitudes towards ACTs and RDTs as part of their routine programme evaluation.

### Study limitations

This study was conducted to explore issues around acceptability and adherence to the new diagnostic and treatment regime in SI. As with the nature of qualitative research, the results are limited in their ability to be generalized to the wider population. However, although the above results reflect community and healthcare worker perceptions in one urban village and one rural village in one Province, the consistency of themes that emerged with existing published literature suggest that the issues may be more broadly applicable. Another limitation of the study may be the potential loss of nuances that can occur through the direct transcription and translation from Pijin to English by the local research officers. Having local co-investigators review transcriptions assisted in minimising this limitation.

Very few participants reported knowing what RDTs are or had seen or experienced this test prior to the study. Therefore, acknowledged as a potential limitation is the argument that notional acceptability is not necessarily congruent with actual acceptability following the use of a particular tool or intervention [31]. Finally, the participants of this study were primarily of the South Sea Evangelical Church, and it is hence unclear whether participants with other religious affiliation would have differing attitudes regarding the acceptability and use of RDTs and ACT.

## Conclusions

This study has identified key issues around acceptability and compliance of using RDTs and ACT in Malaita Province, Solomon Islands. These issues have guided recommendations for the essential next steps to improve compliance and confidence in the new diagnostic and treatment interventions recently implemented in SI. Recommendations include intensive health education to inform communities about the new treatment regime, as well as effective refresher training of healthcare workers, with follow-on activities such as job aides and supportive supervision on the use of RDTs. Attention will also need to be focussed on reducing barriers to early treatment-seeking. Other studies have been undertaken in the country to explore in more depth health seeking behaviours for fever and malaria to complement this study and its findings [32]. It will be essential to elicit the support of influential community members and engage them in health promotion activities. In particular, a participatory action-orientated approach will assist in building partnerships with communities and engage them in the process of local decision-making and implementation of the new treatment regime. In addition, supply lines should be streamlined for RDTs and Coartem^® ^to ensure consistent stocks in the healthcare centres.

Practices such as presumptive treatment and the taking of sub-curative doses are of considerable concern for both the health of individuals and the increased risk it poses to the development of parasite resistance to this important first-line treatment against malaria. Exploring the extent of these issues beyond the study populations must be a priority for malaria programme managers. These recommendations will be integral to the success and sustainability of intensified malaria control, subsequently leading to elimination of malaria in Solomon Islands.

## Competing interests

The authors declare that they have no competing interests.

## Authors' contributions

RW, MW, JA, AB and LW participated in the conception of study design. Training of field researchers in qualitative methods and logistics was carried out by JA and RW. The field research activities were supported and supervised by RW, JA and MW. Data analysis was done by JA with feedback from AB, LW and MW. Manuscript drafting was carried out by RW and JA with reviews and contributions from MW, AB and LW. All authors have read and approved the final manuscript.
